# Silicon Nanowires for Solar Thermal Energy Harvesting: an Experimental Evaluation on the Trade-off Effects of the Spectral Optical Properties

**DOI:** 10.1186/s11671-015-1209-4

**Published:** 2016-01-04

**Authors:** Abdoul Karim Sekone, Yu-Bin Chen, Ming-Chang Lu, Wen-Kai Chen, Chia-An Liu, Ming-Tsang Lee

**Affiliations:** Department of Mechanical Engineering, National Chung Hsing University, Taichung, Taiwan Republic of China; Department of Mechanical Engineering, National Cheng Kung University, Tainan, Taiwan Republic of China; Department of Mechanical Engineering, National Chiao Tung University, Hsinchu, Taiwan Republic of China

**Keywords:** Silicon nanowire, Black silicon, Solar thermal energy harvesting, Reflectivity, Absorption, Transmittance

## Abstract

Silicon nanowire possesses great potential as the material for renewable energy harvesting and conversion. The significantly reduced spectral reflectivity of silicon nanowire to visible light makes it even more attractive in solar energy applications. However, the benefit of its use for solar thermal energy harvesting remains to be investigated and has so far not been clearly reported. The purpose of this study is to provide practical information and insight into the performance of silicon nanowires in solar thermal energy conversion systems. Spectral hemispherical reflectivity and transmissivity of the black silicon nanowire array on silicon wafer substrate were measured. It was observed that the reflectivity is lower in the visible range but higher in the infrared range compared to the plain silicon wafer. A drying experiment and a theoretical calculation were carried out to directly evaluate the effects of the trade-off between scattering properties at different wavelengths. It is clearly seen that silicon nanowires can improve the solar thermal energy harnessing. The results showed that a 17.8 % increase in the harvest and utilization of solar thermal energy could be achieved using a silicon nanowire array on silicon substrate as compared to that obtained with a plain silicon wafer.

## Background

Solar power is the most abundant and intensive source of renewable energy. The global installation of solar-related energy conversion systems keeps increasing rapidly in recent years [1]. There are many ways of utilizing this energy, including photovoltaic, solar-biologics, and photoelectrochemical cells and the harvest of solar thermal energy. Among these, solar thermal systems are simple, efficient, and cost-effective. Solar thermal energy devices include Rankine cycle engines [2], solar dryers [3], solar thermoelectric generators [4], solar thermal reformers [5, 6], and solar water heaters [7]. Nanomaterials, furthermore, have many unique physical properties that make them very attractive and useful in the conversion and utilization of solar energy [8, 9]. Most of the research efforts up to date have been focused on photovoltaics and photocatalysis [10–16], and only a few nanomaterials have been applied in solar thermal energy conversion systems [4, 17–19]. In addition, silicon nanowire has been reported as a promising functional nanomaterial in transistors [20], sensors [21], batteries [22, 23], thermoelectrics [24], photocatalysis [25], piezoelectrics [26], photoelectrochemical cells (PEC) [27], and photovoltaics [14, 28, 29] owing to its preferable optical, thermal, chemical, and mechanical properties in energy conversion systems as well as the fact that silicon nanowire arrays are easy to synthesize [30–32]. Micro/nanoscale-textured silicon surface had been recognized as a potential material for solar thermal energy harvesting [33]. However, despite the well-known feature of the “black silicon” for silicon nanowires [34, 35], there is still a scarcity of reported experimental studies on silicon nanowires for solar thermal energy systems, especially on the spectral-dependent radiative properties and the corresponding solar to thermal energy conversion efficiency. In addition, although silicon nanowire array absorbs visible light more effectively, the trade-off effects of scattering properties at different wavelengths in the vis-near IR range of silicon nanowires have not been clearly investigated and reported. Solar irradiation in the IR range, however, poses a significant fraction of the solar thermal energy that can be utilized in practice. Therefore, in this study, we carried out experimental and theoretical analyses on the solar thermal radiation of the silicon nanowire array radiative properties. Also, to demonstrate its potential application for solar thermal systems where a selective solar spectrum would be required, we conducted solar drying experiments which involved the infrared heating of a moist porous material. The main purpose of this study is to investigate the enhancement of solar thermal energy harvest by silicon nanowire structures where the spectral optical properties including light absorption and scattering vary in the main wavelength range of solar irradiation. The intrinsic optical properties of the thin silicon plates (with or without silicon nanowires) used in these experiments block most ultraviolet and visible light while allowing a significant fraction of infrared to be transmitted, which is assumed to be absorbed directly by the moist porous material below. Based on our literature review, the investigation specifically on the trade-off spectral-dependent solar thermal behavior has not been clearly reported in scientific articles. Therefore, it is emphasized that the results of this study can be used to evaluate the overall solar energy conversion efficiency of silicon nanowire-based hybrid energy conversion systems such as photovoltaic and solar thermal systems.

## Methods

**Fig. 1:**
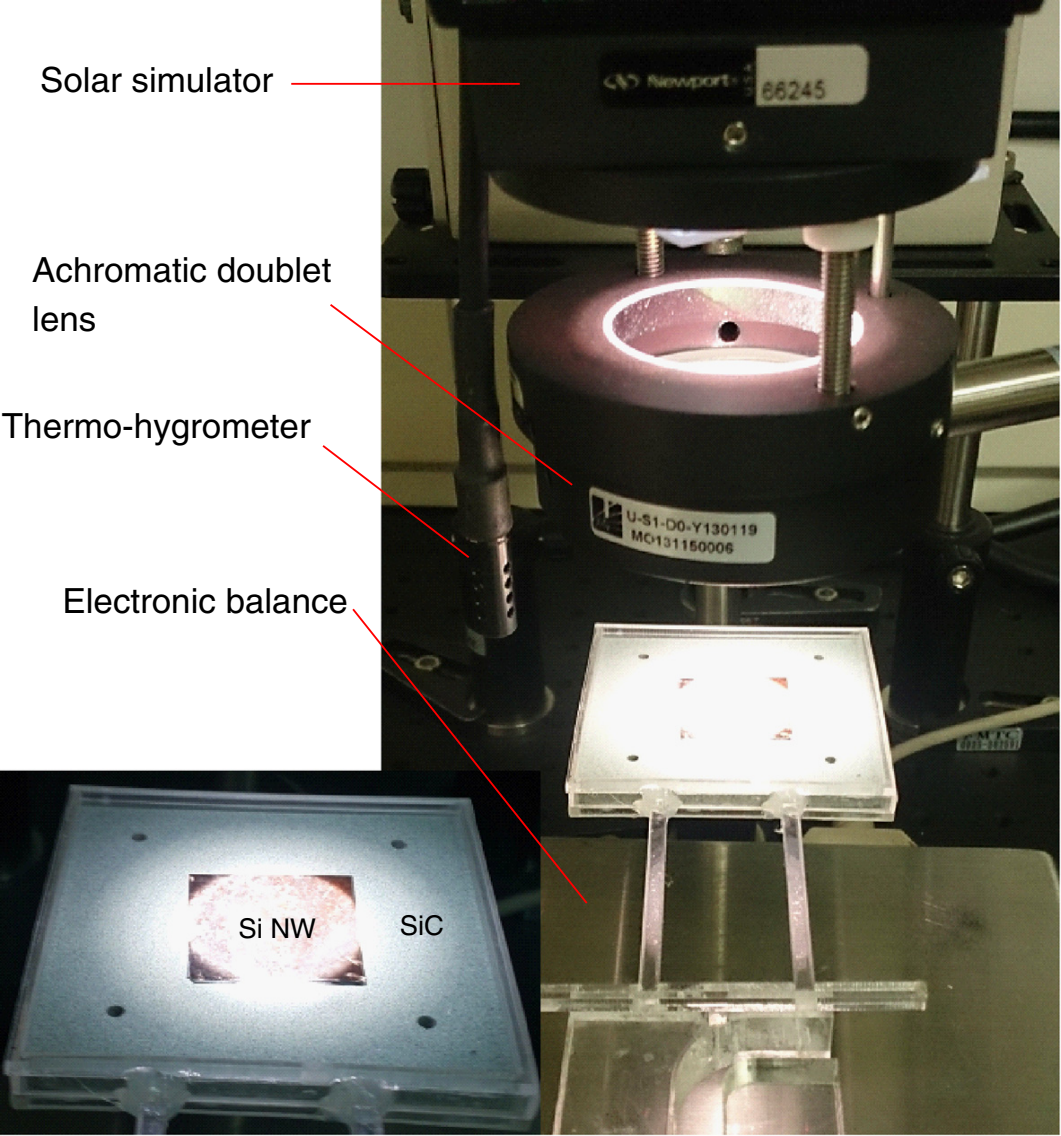
Experimental apparatus of drying experiment

Figure 1 shows the apparatus used for the solar drying experiment. A thin porous silicon carbide (SiC) plate (Silon Sink CYS-516-A) held in a lab-made sample holder, as shown in the inset of Fig. 1, was used for drying measurements. The original mass of the porous plate (size 56.4 × 56.6 × 2.5 mm) was 14.20 g, and its porosity was 29 %. K-type thermocouples (temperature range −270 to 1260 °C, accuracy ±2.2 °C) were used to measure the air temperature at locations near the center on the upper and lower surfaces of the plate as shown in Fig. 1. The relative humidity and temperature of the environment were measured using a thermo-hygrometer (SATO Data Logger®, SK-L200TH II, temperature range −10 to 60 °C, accuracy ±0.1 °C, and ±0.1 % RH). The ambient laboratory temperature was 23 to 25 °C with a relative humidity of 30 to 50 %. Each set of experiments was repeated five times to ensure the accuracy and consistency of the experimental data. An electronic balance (model UW6200HV, Shimadzu Corporation^®^, accuracy ±0.02 g) was used to measure the mass changes of the plate. The plate was fully saturated with distilled water before the drying experiment was started. More details of the drying experimental procedure can be found in a previously reported study [36]. The experiments included both natural drying and solar drying procedures. A shield made of acrylic plates was applied to minimize the impact from the surroundings. For natural drying, the SiC plate was simply allowed to dry in the laboratory environment. A solar light simulator (Newport 96000 Solar Simulator with an AM 1.5 filter) was used to simulate solar irradiation. The intensity of the solar light used was 1 kW/m^2^ which corresponded to 1 sun. The diameter of the original beam of the simulated solar light is 5 cm and was concentrated using a visible light achromatic doublet lens (Thorlabs Inc., focal length *f* = 75 mm) to produce a light spot 2 cm in diameter.

**Fig. 2:**
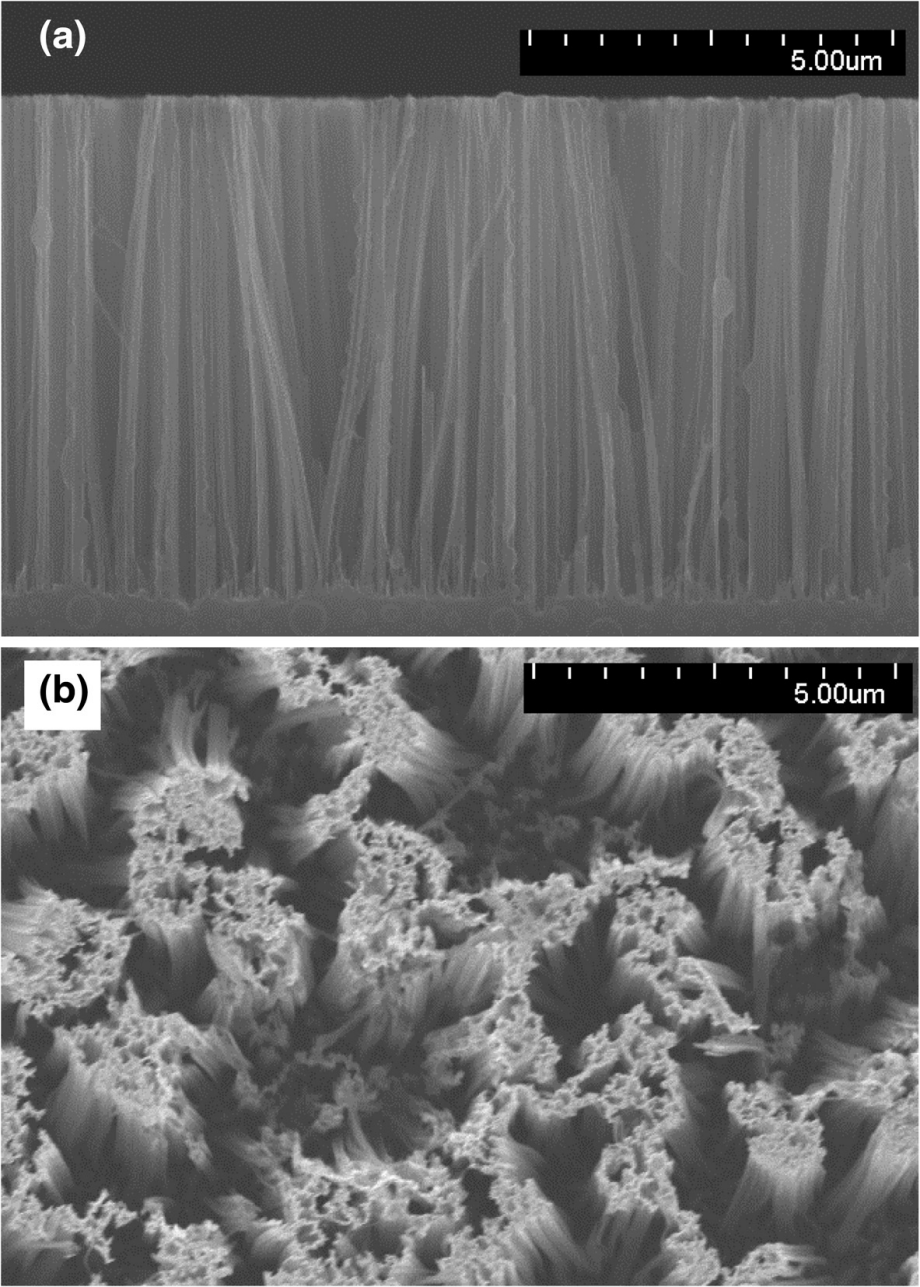
SEM image of silicon nanowires. **a** Side view. **b** Top view

The silicon nanowires were synthesized by a wafer-scale aqueous electroless etching technique [37]. Briefly, a silicon wafer (thickness 500 μm) was immersed into an aqueous solution of AgNO_3_ to oxidize the silicon lattice and this was followed by HF acid etching. The un-etched silicon formed nanowires. Figure 2 is a SEM image of the nearly vertical-aligned silicon nanowires. These synthesized nanowires have diameters in the range of 50 to 300 nm and are approximately 7 μm high. The spectral hemispherical reflectivity (R) and transmissivity (T) of the silicon and silicon nanowire plates were measured using a hemispherical radiative property measurement system (range 400–1800 nm, spectral resolution 20 nm) [38]. The incident angle was 5° from normal to the sample surface. The hemispherical measurement system was calibrated and validated by the measured scattering properties of a standard planar silicon wafer. Details of the spectral scattering property experiment can be found in a previously reported study by Chen et al. [38]. It should be noted that the non-vertical nanowires will affect the optical properties of the silicon nanowire bundle. For example, the corrugated surface may re-absorb the reflected and emitted irradiation between adjacent surfaces, which is referred to the multiple scattering or light-trapping effect [35]. In addition, the diameter of the silicon nanowires varies between 50 and 300 nm as previously noted. The resonance on the optical properties of the silicon nanowire is also affected by its diameter. In general, a wide range of diameters yields a broadened spectral resonance. However, in this study, we measured the optical properties (absorptivity, reflectivity, and transmissivity) directly on the silicon nanowire bundles. Therefore, the following theoretical analysis based on these experimental measurements had already taken the light-trapping effect resulted into account.

## Results and Discussion

**Fig. 3:**
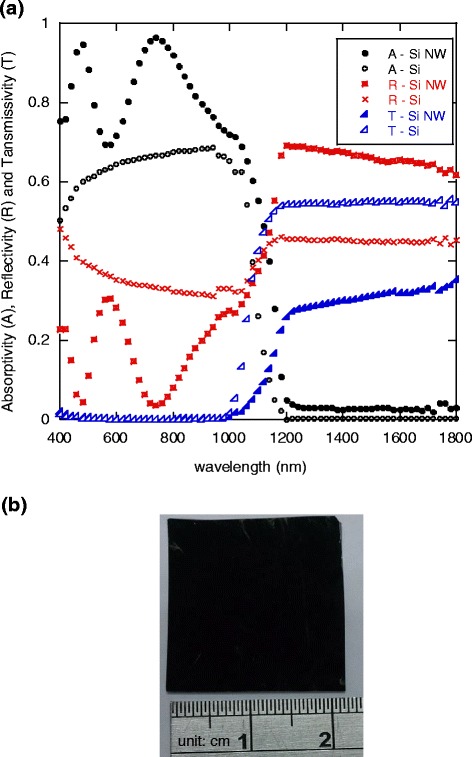
**a** Measured spectral absorptivity (*A*), reflectivity (*R*), and transmissivity (*T*) of plain silicon substrate (*Si*) and silicon nanowires (*Si NW*). **b** Black silicon nanowire array plate fabricated and used for the drying experiment

Results of the scattering measurements are shown in Fig. 3. The absorptivity (A) of the silicon and silicon nanowire plates can be obtained from the measurements since summation of A, T, and R should equal unity. The results for the spectral absorptivity are shown in Fig. 3. Figure 3b is an image of the silicon nanowire array. The black color appearance of the surface indicates that it is much more absorptive than that of the typical silver-gray planar silicon surface, and is promising for the effective absorption of solar irradiation, especially of visible wavelength [34, 35]. Silicon nanowires easily form a native silicon dioxide layer on the surface. The native silicon dioxide layer tends to absorb infrared. This might explain the slightly increased absorptivity of silicon nanowire surface compared to that of the plain silicon in the infrared range. However, it is also noticed that in the infrared range, the reflectivity of the silicon nanowires increases. This might be attributed to the fact that the silicon nanowire surface is optically smooth to the long wavelength irradiation since the diameters of silicon nanowires are in the range of 50 to 300 nm, and the length of the nanowires is not sufficiently long to effectively increase the light-trapping effect. The increased surface area of the nanowires thus increases the reflectivity. This result might lead to a reduction in overall solar irradiation harvest, especially important in solar thermal applications. Therefore, to evaluate the trade-off effects of the changes in scattering and absorption properties, we performed drying measurements to directly investigate and compare the performance of solar thermal energy harnessing and conversion between the silicon wafer plates with and without silicon nanowire array on the surface facing to the incident solar light.

**Fig. 4:**
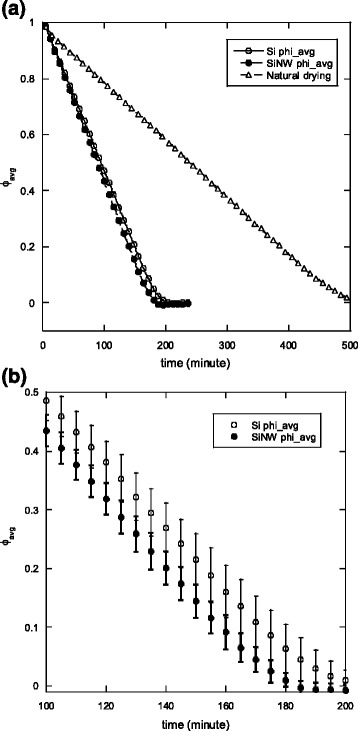
Variation of the dimensionless average moisture content in the SiC plate with respect to drying time. **a** Complete drying curve. **b** Drying curves for silicon and silicon nanowire cases between 100 and 200 min

Figure 4a shows the results for the changes in the average dimensionless moisture content of the plate (*ϕ*_avg_) during the drying process which can be defined as

1 $$ {\phi}_{\mathrm{avg}}=\frac{m\left(\mathrm{t}\right)-{m}_{\mathrm{e}}}{m(0)-{m}_{\mathrm{e}}} $$

where *m*(t) is the mass of the SiC plate at a given time and *m*_e_ is the mass of the dry SiC plate. The initial mass of the wet SiC plate is approximately 18.1 to 18.5 g. To demonstrate the results more clearly for a better comparison, the drying curves of plain silicon and silicon nanowire plates are shown in detail for drying times between 100 and 200 min (with error bars) in Fig. 4b. Note that the air temperatures near the top and the bottom surfaces during the drying experiments are similar, i.e., no significant temperature differences between each other, and varied accordingly with the variation of the room temperature (within about 2 °C). Therefore, the differences on the drying rate due to the environment temperature variation are assumed to be negligible. It can be seen from Fig. 4 that the drying rate of the porous SiC plate with a silicon nanowire plate on the top surface is faster than that with a plain silicon plate. The energy of the incident solar light can be approximated by the Plank distribution for blackbody:

2 $$ {E}_{\lambda,\ b}=\frac{C_1}{\lambda^5\left[ \exp \left({C}_2/\lambda T\right)-1\right]} $$

where *λ* is the wavelength (unit μm), *C*_1_ = 3.742 × 10^8^ W·μm^4^/m^2^, *C*_2_ = 1.439 × 10^4^ μm·K, and *T* = 5777 K [39]. From Eq. (2) and the measured absorptivity and transmissivity as shown in Fig. 3, the incident solar energy being absorbed (*E*_abs_) and transmitted (*E*_trans_) through the plain silicon and silicon nanowire plates for wavelengths between 0.4 and 1.8 μm can be calculated from

3 $$ {E}_{\mathrm{abs}}={\displaystyle {\int}_{0.4}^{1.8}{\alpha}_{\lambda }{E}_{\lambda, b}d\lambda } $$

4 $$ {E}_{\mathrm{trans}}={\displaystyle {\int}_{0.4}^{1.8}{\tau}_{\lambda }{E}_{\lambda, b}d\lambda } $$

5 $$ {E}_{\mathrm{refl}}={\displaystyle {\int}_{0.4}^{1.8}{\rho}_{\lambda }{E}_{\lambda, b}d\lambda } $$

where *α*_*λ*_, *τ*_*λ*_, and *ρ*_*λ*_ are the spectral absorptivity, transmissivity, and reflectivity, respectively. Note that this wavelength interval was decided according to the range of the hemispherical radiative property measurement system as previously indicated. In addition, this wavelength range encompasses the main fraction of solar spectra. It is assumed that the solar energy transmitted through the silicon plate (with or without silicon nanowires) can be effectively absorbed by the moist porous SiC plate considering its thickness (2.5 mm) and the porous structure. It should be pointed out that, although SiC crystal has small optical absorption coefficient in general [40, 41], the porous structure and impurities in the SiC plate used in the current study may result in high absorptivity to the wavelength of interests. Thus, the incident solar energy, except the fraction being reflected by the cover plate surface, was assumed to be absorbed by the porous SiC plate and the silicon plate on top. The ratio of the solar irradiation that can be utilized by the current configuration of the drying system can therefore be obtained:

6 $$ \eta =\frac{E_{\mathrm{abs}}+{E}_{\mathrm{trans}}}{E_{\mathrm{abs}}+{E}_{\mathrm{trans}}+{E}_{\mathrm{refl}}} $$

where the denominator in Eq. (6) is equivalent to the blackbody solar irradiation for wavelength in the range from 0.4 to 1.8 μm. Results of absorbed, transmitted, and reflected solar irradiation energy of silicon and silicon nanowires are summarized in Table 1. It should be pointed out again that the reflectivity of silicon nanowire surface for wavelengths between 0.4 and 1.1 μm is significantly lower than that of a plain silicon surface. However, in the range between 1.1 and 1.8 μm, the silicon nanowire plate showed slightly greater reflectivity. Therefore, to evaluate and compare the total energy utilized by the silicon and silicon nanowire plates, one should consider the spectral radiative properties (reflectivity, transmissivity, and absorptivity) in conjunction with the solar irradiation spectrum. From Table 1, it can be seen that the harvest and utilization of solar thermal energy achieved by the silicon nanowire is 73.5 %, which is apparently better than that of the plain silicon plate (62.4 %). The ratio of the solar irradiation utilization (*η*) increased by 17.8 %. This is also the reason why the drying rate of the porous SiC with the silicon nanowire plate on top was faster. These results lead to the conclusion that the increased absorptivity, and subsequent harnessing of solar energy, of the silicon nanowires in the visible range adequately compensates for the increased reflectivity in the infrared. The absorptivity of the silicon nanowire bundles to visible light can be further increased by increasing the length of the wires taking the advantage of multiple reflection and absorption in the nanowire array as previously noted [35]. Furthermore, the reflectivity of the silicon nanowire arrays in the ultraviolet and infrared range could be significantly reduced with heterogeneous and hierarchical material coatings such as of zinc oxide [42] which would make the silicon-based nanostructures more efficient for the purpose. As previously discussed, the nanostructure has been proven to effectively improve the efficiency of silicon-based photovoltaic systems. However, the benefits of the increased solar thermal energy harvesting from silicon nanowires for thermal energy conversion systems remained to be discovered. Clearly, the results of this study can be utilized in the evaluation of hybrid solar energy systems [43] such as silicon-based solar cells and solar heaters with silicon nanostructures to improve photovoltaic and solar thermal efficiency simultaneously.

**Table 1:** Absorbed, transmitted, and reflected solar irradiation of silicon and silicon nanowires (between 0.4 and 1.8 μm)

	*E*_abs_ × 10^−7^ (W/m^2^)	*E*_trans_ × 10^−7^ (W/m^2^)	*E*_refl_ × 10^−7^ (W/m^2^)	*η* (%)
Si	2.62	0.60	1.94	62.4
Si NW	3.48	0.29	1.36	73.5

## Conclusions

In this study, the performance of silicon nanowires for the harnessing of solar thermal energy was evaluated experimentally. Measurements of the optical properties of silicon nanowire arrays were conducted. Drying experiments of a porous SiC plate were carried out to investigate the enhancement of the solar thermal energy harvested and utilized by the silicon nanowire arrays. The results showed that the drying rate of the porous SiC plate covered by the silicon nanowire plate was greater than that with the plain silicon. Theoretical estimations of the fraction of solar irradiation energy absorbed by the drying system (SiC plate covered by silicon or silicon nanowire plate) were carried out using the thermal radiation theory and measurement of the optical properties of the silicon and silicon nanowire arrays, respectively. The theoretical results suggest that use of the silicon nanowire array leads to a 17.8 % increase in the overall harvest of solar thermal energy. These results can be utilized to evaluate hybrid solar energy systems such as silicon-based solar cells and solar heaters with silicon nanostructures to improve both the photovoltaic and solar thermal efficiency simultaneously.
